# Depressive symptoms and suicidal behaviours in adolescent non-daily smokers compared to daily smokers and never-smokers in Korea: National cross-sectional study

**DOI:** 10.1371/journal.pone.0207182

**Published:** 2018-11-14

**Authors:** Jinhee Lee, Tae Hui Kim, Seongho Min, Min-Hyuk Kim, Ki Chang Park, Jin Sil Moon, Joung-Sook Ahn

**Affiliations:** 1 Division of Child and Adolescent Psychiatry, Yonsei University Wonju College of Medicine, Wonju, Korea; 2 Department of Psychiatry, Yonsei University Wonju College of Medicine, Wonju, Korea; 3 Center of Biomedical Data Science (CBDS), Yonsei University Wonju College of Medicine, Wonju, Korea; University of Toronto, CANADA

## Abstract

**Introduction:**

We aimed to investigate the association of non-daily smoking with depressive symptoms and suicidal behaviours among adolescents by analysing data from the 2016 Korean Youth Risk Behavior Web-based Survey (KYRBWS), a national school-based survey.

**Methods:**

We analysed data from a nationally representative sample of Korean adolescents aged 12–18 years (n = 65,528). We investigated the risks of depressive symptoms, suicide ideation, plan and attempt in adolescent non-daily smokers using multiple logistic regression analyses after adjusting for confounding factors. Taking into account the trajectory of smoking patterns in adolescents, we assessed all analyses with stratification by early (aged 12–15) and late (aged 16–18) adolescents.

**Results:**

Among early adolescents, non-daily smokers were more likely to have depressive symptoms, suicide ideation and plan compared with never smokers and even more likely to have depressive symptoms compared with daily smokers. Among late adolescents, non-daily smokers had higher risks of depressive symptoms, suicide ideation, plan and attempt than never smokers, whereas the risk for suicide attempts was lower than daily smokers.

**Conclusions:**

Our findings suggest that non-daily smoking in adolescents was associated with risks for depressive symptoms and suicidal behaviours, and the association was more prominent in early adolescents. Careful attention on the mental health of adolescent non-daily smokers is needed because this is an increasing and easily overlooked group.

## Introduction

The use of tobacco by adolescents is a major public health problem in most regions worldwide [1].

Approximately 80% of tobacco smoking begins in adolescence [2], and patterns of smoking are often established during adolescence [3]. Progressing through the series of smoking stages, such as trying cigarettes, repeated experimentation and dependence, before becoming regular smokers is common in adolescents [4]. Studies indicated that the trajectories of adolescent smoking show that the majority of adolescent smokers begin experimenting by the age of 12 and remain irregular smokers consistently throughout adolescence [4,5], suggesting the importance of the attention to experimental or irregular smokers among adolescents. The regularity of smoking is commonly divided into daily smoking and non-daily smoking [6]. Some previous studies reported that most adolescent smokers are non-daily smokers and their proportion is rapidly increasing [7–9] despite various social pressure and efforts made to delay smoking initiation among adolescents [10]. Non-daily smoking as experimental or intermittent smoking is completely differentiated from addictive smoking by the presence of daily smoking in adolescents [11]. Although the number of health problems associated with smoking is known to vary based on the smoking frequency [12], there is no known safe level of tobacco smoking [13]. However, Non-daily smoking is easily overlooked as less dangerous than daily smoking, and many adolescents believe it will cause little or no harm [14].

Adolescent smoking is associated with a number of physical and psychiatric health problems, such as depression, anxiety or other problematic substance use [15–17]. Tobacco smoking is universally accepted to be associated with depression, and some studies reported that the relationship between smoking frequency and depression was found to vary by age and gender [6,18]. A population study with 78,229 adults found that non-daily smokers differed in mental health behaviours from daily or never smokers [12]. On the contrary, another cross-sectional study reported that smoking frequency was not associated with past‐year major depressive episode after adjusting for socio-demographics and health confounders [6]. With regard to the age difference, the most recent longitudinal study on Americans reported that the incidences of depression seem to be increasing over time in non-daily smokers, especially among youth [8]. With regard to the gender difference, another research suggested that depression is associated with non-daily smoking in women but not in men [18]. Although a number of studies investigated the relationship of smoking frequency with depression in adults [8] or the relationship between smoking and depression in adolescents [19–21], very few studies investigated the association of non-daily smoking and mental health problems in the general adolescent population aged 12–18 years.

Furthermore, depression is one of the most significant risk factors for suicide, and the adolescence period is known to have a higher vulnerability to suicide [22]. Although several studies have been conducted on suicidal behaviour and adolescent tobacco smoking [23–25], generalisation is limited and very few studies have attempted to examine the relationship between smoking frequency and suicidal behaviours. Considering the fact that suicidal behaviours of adolescents are a major public health problem in many countries recently, clarifying the details of smoking related to suicidal behaviours is necessary.

Taking into account all these aspects, it is necessary to evaluate and understand the depressive symptoms and suicidal behaviours of adolescent non-daily smokers, whose risk is easily overlooked. The present study involved a large, representative, cross-national sample of school-based adolescents in Korea. The main objectives of this study were as follows: (1) to assess the influence of socio-demographic factors and smoking-related factors on Non-daily smoking in adolescents compared with Daily smoking or Never-smoking and (2) to investigate the association between Non-daily smoking and depressive symptoms and suicidal behaviours (suicide ideation, plan and attempt).

## Methods

### Study population and source of data

We analysed data from the 12th Korea Youth Risk Behavior Web-based Survey (KYRBS) administered in 2016 by the Korean Ministry of Education, Science and Technology, Ministry of Health and Welfare and Korea Centers for Disease Control and Prevention (KCDC) in this study. KYRBS is a self-reported anonymous online survey, which uses a nationally representative sample of Korean adolescents (aged 12–18 years). The sample design of this survey used a stratified multistage cluster strategy with 129 questions divided into 15 sections inquiring about health-related behaviours, mental and physical health. In the twelfth KYRBS, 67,983 students from 800 middle and high schools were randomly selected, and 65,528 (33,803 boys and 31,725 girls) students (96.4% response rate) from 798 schools (99.8% response rate) responded to the survey [26]. Participants were provided with identification numbers and were guaranteed anonymity, and all participants completed an online, self-reported questionnaire in a school computer room after the survey had been fully explained. All data used in this study have been fully anonymized before we accessed them. KYRBS was approved by the Institutional Review Board of the Korea Centers for Disease Control and Prevention.

### Questionnaire

Baseline evaluations were conducted in June 2016 and included items on socio-demographic factors, smoking-related factors, depressive symptoms and suicidal behaviours.

#### Smoking frequency

We assessed the cigarette smoking frequency of the subjects to evaluate the smoking pattern of adolescents. The cigarette smoking frequency was assessed using the following questions: (1) ‘Have you ever smoked part or all of a cigarette? (Yes/No)’ (2) ‘How many days during the past 30 days did you smoke a cigarette? (None/ 1–2 days/3–5 days/ 6–9 days/10–19 days/20–29 days/every day)’. Respondents who responded to ‘No’ to the first question were classified as never smokers (NS). Respondents who responded ‘Yes’ to the first question and answered ‘Every day’ to the second question were classified as daily smokers (DS). Respondents who responded to ‘Yes’ to the first question and responded from ‘1–2 days’ to ‘20–29 days’ were classified as non-daily smokers (NDS). We excluded 5,499 (8.4%) respondents from the analysis who stated that they experienced smoking during their lifetime but did not smoke a cigarette in the past month (e.g., ex-smokers) [8]. Finally, we divided the subjects into the following three groups: Never smokers (NS; n = 56,017 (93.3%)), Daily smokers (DS; n = 1,903 (3.2%)) and Non-daily smokers (NDS; n = 2,109 (3.5%)).

#### Socio-demographic characteristics

Socio-demographic characteristics included age, sex, residential area, family economic status, paternal and maternal education levels and academic achievement of the participant. Respondents who lived in the county or rural areas were categorised as ‘Rural’; those who lived in small, middle-sized or large cities were categorised as ‘Urban’. Family economic status was assessed by the question, ‘What is your family economic status’? The five possible response categories very high/high/middle/low/very low were grouped into three categories as high (very high or high), middle (middle) and low (very low or low) [27]. Paternal and maternal education levels were assessed with the following options: ‘less than or equal to middle school graduate’, ‘high school graduate’, ‘more than or equal to college graduate’, ‘don’t know’ and ‘not applicable (in case the participant’s parents were deceased)’. We reclassified the measures into three categories as ‘less than or equal to high school graduate’, ‘more than or equal to college graduate’ and ‘unknown (do not know or not applicable)’. Academic achievement was assessed by asking ‘During the past 12 months, how would you rate your academic performance’? The five possible responses categories high/middle–high/middle/middle–low/low were grouped into three categories as high (high or middle–high), middle (middle) and low (middle–low or low) [28]. We also evaluated some types of adolescent mental health-related factors, such as sleep satisfaction, current alcohol drinking and experience of violence that are well-known critical factors for developing depressive feelings and suicidal behaviours in adolescents [29–31]. Perceived sleep satisfaction was measured by the question ‘In the last week, how satisfactory was your sleep in terms of relieving your fatigue’? with response options of ‘very unsatisfactory’, ‘unsatisfactory’, ‘average’, ‘satisfactory’ and ‘very satisfactory’. The answers were reclassified into three categories: ‘unsatisfactory (very unsatisfactory or unsatisfactory)’, ‘average (average)’ and ‘satisfactory (satisfactory or very satisfactory)’. Current alcohol drinking was assessed by the question ‘How many days during the past 30 days did you drink more than one cup of alcohol? (None/1–2 days/3–5 days/6–9 days/10–19 days/20–29 days/Every day)’ Respondents who responded ‘None’ were classified as not current alcohol drinkers and those who responded ‘1-2days’ to ‘Every days’ were classified as current alcohol drinkers. Respondents were categorised as having experienced violence if they reported undergoing treatment at hospital physical and/or psychological violence, such as bullying or threatening by others at least once during the last 12 months.

#### Cigarette smoking-related factors

Environmental factors that could affect adolescent cigarette smoking and characteristics of smoking behaviours had been assessed [32–34]. Second-hand smoke exposure in the household was assessed by asking ‘How many days during the last 7 days were you near when your family members were smoking at home? (None/1 day/2 days/3 days/4 days/5 days/6 days/Every day)’. Respondents who responded ‘None’ were classified as not second-hand smokers, and those who responded from ‘1 day’ to ‘Every day’ were classified as second-hand smokers. Friends’ smoking status was assessed by the question ‘Do any of your close friends smoke? (None/Some friends/Most friends/All friends)’. The answers were reclassified into three categories: none, some and most/all. The first experience of smoking was assessed with the following options: ‘before or during elementary school’ and ‘during in middle or high school’. Smoking volume was measured using a graded categorical variable coded from 1 to 3: 1 (smoked 1–9 cigarettes), 2 (smoked 10–19 cigarettes) and 3 (smoked 20 or more cigarettes). Any attempt to stop smoking of subjects was assessed by asking ‘Have you ever try to quit smoking during the past 12 months?’ (Yes/No).

### Depressive symptoms and suicidal behaviour

#### Depressive symptoms

Depressed mood among the subjects was assessed by the question ‘In the past year, have you ever felt sad or despaired that your feelings disturbed everyday life for whole two weeks’? Subjects responded with the following: 1) No, I never felt it or 2) Yes, I have felt it.

#### Suicide ideation

We examined whether the subjects had suicidal ideations with the question ‘In the past year, did you ever seriously consider attempting suicide’? Subjects responded with the following: 1) No, I never thought of it or 2) Yes, I have thought of it.

#### Suicide plan

Suicide plan among the subjects was assessed by the question ‘In the past year, have you ever had a specific plan to attempt suicide’. Subjects responded with the following 1) No, I never planned to it or 2) Yes, I have planned to it.

#### Suicide attempt

Attempted suicide was defined as a positive response to the following question ‘In the past year, have you ever attempted suicide’? Subjects responded with the following: 1) No I never attempted suicide or 2) Yes, I have attempted suicide.

### Statistical analyses

Univariate analysis was conducted to estimate the proportion of each smoking pattern among Korean adolescents. Pearson’s chi square test was carried out to estimate the socio-demographic characteristics and smoking-related factors of the three groups. The relationship of the three different groups with depressive symptoms and suicidal behaviours was analysed using Pearson’s chi square, and subsequently, multiple logistic regression analysis was performed to identify the association (NDS vs. NS; DS vs. NS; NDS vs. DS). Socio-demographic, mental health-related factors and smoking-related factors that showed a significant difference in the Chi-square test were mutually adjusted for the analysis. In addition, the results of the multivariable logistic regression model, which included depressive symptoms and suicidal behaviours as independent variables with other potential confounders, were presented. Considering the various trajectories of adolescent smoking [4,35] and the different associations between smoking frequency and mental health outcomes with respect to age in a previous study [36], the analysis were assessed after stratifying the subjects by age group; Early adolescents (EA, aged 12–15 years; mean age, 13.52 years (SD = 0.95) and Late adolescents (LA, aged 16–18 years; mean age, 16.42 years (SD = 0.96). The results were reported as adjusted odds ratios (aORs) with 95% confidence intervals (CIs) and *p* values. All *p* values <0.05 were considered to be statistically significant. All statistical analyses were performed using SPSS (version 23.0, IBM Corp., Armonk, NY, USA).

## Results

### Socio-demographic factors and smoking-related factors of subjects

**Fig 1:**
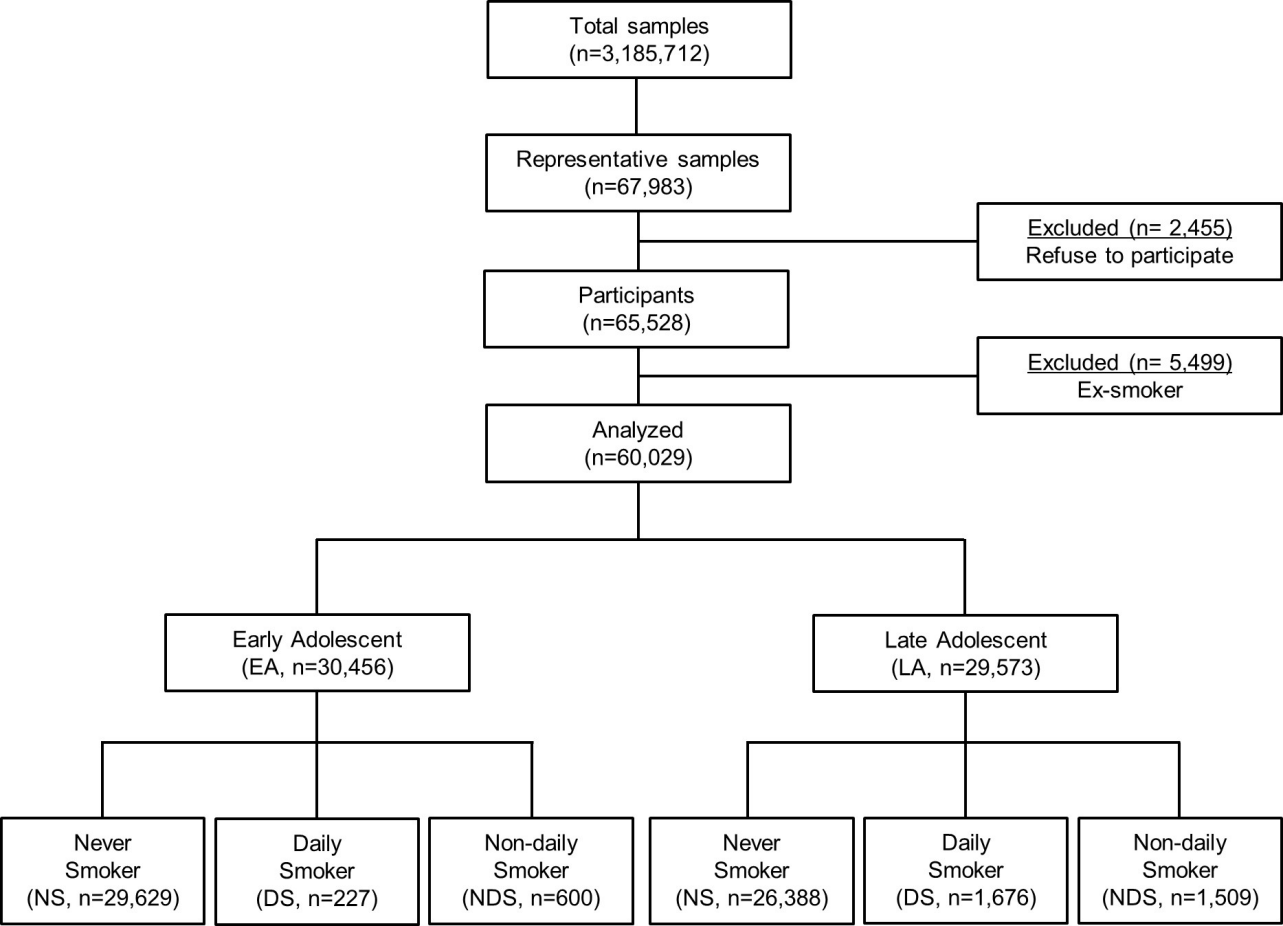
STROBE diagram for the flow of present study.

The final sample of 60,029 adolescents was divided into three groups based on smoking frequency. Fig 1 shows the STROBE diagram for the flow of the present study. In this representative study population, 3.17% (0.74% for EA and 5.66% for LA) and 3.51% (1.97% for EA and 5.10% for LA) of Korean adolescents reported themselves as DS and NDS, respectively. The socio-demographic characteristics of the NS, DS and NDS are described in Table 1. Results of the Chi-square analysis revealed that the adolescents showed significant differences depending on their sex, residential area, family economic status, paternal and maternal education levels, academic achievement, sleep satisfaction, current alcohol drinking and experience of violence by smoking status. Among EA, NDS were more likely to have high family economic status (40.0%, *p* < 0.001), whereas among LA, NDS was more likely to have middle family economic status (44.2%, *p* < 0.001). In EA, NDS had a higher prevalence of high paternal education level, college diploma or more than DS (NDS: 31.7% vs. DS: 30.8%, *p* < 0.001), but in LA, NDS had a lower prevalence of high paternal education level than DS (NDS: 39.0% vs. DS: 42.6%, *p* < 0.001). Both NDS in EA and LA were more likely to have lower prevalence of high maternal education level than DS (NDS: 32.3% vs. DS: 35.2% in EA, *p* < 0.001; NDS: 43.4% vs. DS: 46.7% in LA, *p* < 0.001). Among EA, NDS had a lower prevalence of high academic achievement than DS (NDS: 24.7% vs. DS: 29.5%, *p* < 0.001), but among LA, NDS had a higher prevalence of high academic achievement than DS (NDS: 25.6% vs. DS 22.7%, *p* < 0.001). NDS in EA were less likely to consume alcohol currently (49.2% of NDS), whereas NDS in LA were more likely to consume alcohol (65.5% of NDS). In EA, NDS had a lower prevalence of experiencing violence than DS (NDS: 11.0% vs. DS: 18.5%, *p* < 0.001), whereas in LA, NDS had a higher prevalence of experience of violence than DS (NDS: 11.4% vs. DS: 7.1%, *p* < 0.001)

**Table 1:** Socio-demographic characteristics based on smoking patterns (KYRBS, 2016, Korean adolescents aged 12–18 years).

	Early adolescents (n = 30,456)				Late adolescents (n = 29,573)			
	Never smokers (n = 29,629) n (%)	Daily smokers (n = 227) n (%)	Non-daily smokers (n = 600) n (%)	p-value	Never smokers (n = 26,388) n (%)	Daily smokers (n = 1676) n (%)	Non-daily smokers (n = 1,509) n (%)	p-value
**Age** **(mean ± SD)**	13.48 ± 0.96	14.45 ± 0.80	13.99 ± 0.95	<0.001	16.39 ± 0.95	16.74 ± 0.90	16.43 ± 0.98	<0.001
**Sex**				<0.001				<0.001
Male	14,774 (49.9)	173 (76.2)	440 (73.3)		11,761 (44.6)	1,418 (84.6)	1,127 (74.7)	
Female	14,855 (50.1)	54 (23.8)	160 (26.7)		14,627 (55.4)	258 (15.4)	382 (25.3)	
**Residential Area**				0.004				<0.001
Urban	14,966 (50.5)	129 (56.8)	270 (45.0)		14,059 (53.3)	821 (49.0)	724 (48.0)	
Rural	14,663 (49.5)	98 (43.2)	330 (55.0)		12,329 (64.7)	855 (51.0)	785 (52.0)	
**Family Economic Status**				<0.001				<0.001
High	12,780 (43.1)	93 (41.0)	240 (40.0)		8,337 (31.6)	549 (32.8)	455 (30.2)	
Middle	13,636 (46.0)	72 (31.7)	237 (39.5)		13,231 (51.0)	666 (39.7)	667 (44.2)	
Low	3,213 (10.8)	62 (27.3)	123 (20.5)		4,820 (18.3)	461 (27.5)	387 (25.6)	
**Paternal Education**				<0.001				<0.001
High school	7,122 (24.0)	70 (30.8)	190 (31.7)		8,905 (33.7)	714 (42.6)	589 (39.0)	
≥ College	14,507 (49.0)	76 (33.5)	227 (37.8)		13,573 (51.4)	600 (35.8)	609 (40.4)	
Unknown	8,000 (27.0)	81 (35.7)	183 (30.5)		3,910 (14.8)	362 (21.6)	311 (20.6)	
**Maternal Education**				<0.001				<0.001
≤ Highschool	8,519 (28.8)	80 (35.2)	194 (32.3)		10,987 (41.6)	783 (46.7)	655 (43.4)	
≥ College	13,654 (46.1)	73 (32.2)	226 (37.7)		11,732 (44.5)	529 (31.6)	526 (34.9)	
Unknown	7,456 (25.2)	74 (32.6)	180 (30.0)		3,669 (13.9)	364 (21.7)	328 (21.7)	
**Academic Achievement**				<0.001				<0.001
High	12,821 (43.3)	67 (29.5)	148 (24.7)		9,743 (36.9)	381 (22.7)	386 (25.6)	
Middle	8,129 (27.4)	36 (15.9)	130 (21.7)		8,106 (30.7)	367 (21.9)	410 (27.2)	
Low	8,679 (29.3)	124 (54.6)	322 (53.7)		8,539 (32.4)	928 (55.4)	713 (47.2)	
**Sleep Satisfaction**				<0.001				0.003
Well enough	10,574 (35.7)	55 (24.2)	171 (28.5)		4,877 (18.5)	276 (16.5)	284 (18.8)	
Enough	10,100 (34.1)	59 (26.0)	206 (34.3)		7,772 (29.5)	444 (26.5)	449 (29.8)	
Not enough	8,955 (30.2)	113 (49.8)	223 (37.2)		13,739 (52.1)	956 (57.0)	776 (51.4)	
**Current Alcohol Drinking**				<0.001				<0.001
No	28,209 (95.2)	753 (33.0)	305 (50.8)		22,615 (85.7)	402 (24.0)	521 (34.5)	
Yes	1,420 (4.8)	152 (67.0)	295 (49.2)		3,773 (14.3)	1,274 (76.0)	988 (65.5)	
**Experience Of Violence**				<0.001				<0.001
Never experienced	29,084 (98.2)	185 (81.5)	534 (89.0)		25,975 (98.4)	1,557 (92.9)	1,337 (88.6)	
Experienced ≥1 time	545 (1.8)	42 (18.5)	66 (11.0)		413 (1.6)	119 (7.1)	172 (11.4)	

Table 2 shows the proportions of cigarette smoking-related factors by smoking frequency of adolescents. The proportion of second-hand smoke exposure in household was higher in NDS than that in DS among both EA (NDS: 52.2% vs. DS: 49.3%, *p* < 0.001) and LA (NDS: 47.0% vs. DS: 43.0%, *p* < 0.001). However, NDS were likely to have less number of friends who smoke than DS in both EA (NDS: 84.3% vs. DS: 92.1%, *p* < 0.001) and LA (NDS: 94.0% vs. DS: 97.1%, *p* < 0.001). NDS in EA were trying lesser to quit smoking during the past year than DS (NDS: 72.5% vs. DS 73.6%, *p* < 0.001), although NDS in LA were trying harder to quit smoking compared with DS (NDS: 71.1% vs. DS: 68.6%, *p* < 0.001).

**Table 2:** Cigarette smoking-related factors based on smoking patterns (KYRBS, 2016, Korean adolescents aged 12–18 years).

	Early adolescents (n = 30,456)				Late adolescents (n = 29,573)			
	Never smokers (n = 29,629) n (%)	Daily smokers (n = 227) n (%)	Non-daily smokers (n = 600) n (%)	p-value	Never smokers (n = 26,388) n (%)	Daily smokers (n = 1,676) n (%)	Non-daily smokers (n = 1,509) n (%)	p-value
**Second-Hand Smoke Exposure In Household (week)**				<0.001				<0.001
No	20,889(70.5)	115(50.7)	287(47.8)		19,382(73.5)	956(57.0)	800(53.0)	
Yes	8,740(29.5)	112(49.3)	313(52.2)		7,006(26.5)	720(43.0)	709(47.0)	
**Friends’ Smoking Status**				<0.001				<0.001
No	23,819 (80.4)	18 (7.9)	94 (15.7)		14,882 (56.4)	48 (2.9)	90 (6.0)	
Yes	5,810 (19.6)	209 (92.1)	506 (84.3)		11,506 (43.6)	1,628 (97.1)	1,419 (94.0)	
**First Experience Of Smoking**				<0.001				<0.001
≤ Elementary school	-	109 (48.4)	209 (35.1)		-	488 (29.2)	349 (23.2)	
≤ Middle/High school	-	116 (51.6)	387 (64.9)		-	1,184 (70.8)	1,156 (76.8)	
**Smoking Volume**				<0.001				<0.001
1–9/day	-	146 (64.3)	551 (91.8)		-	1,120 (66.8)	1,354 (89.7)	
10–19/day	-	46 (20.3)	28 (4.7)		-	376 (22.4)	89 (5.9)	
≥ 20/day	-	35 (15.4)	21(3.5)		-	180 (10.7)	66 (4.4)	
**Trying To Quit Smoking During The Past 12 Months**				<0.001				<0.001
Absent	-	60 (26.4)	165 (27.5)		-	527 (31.4)	436 (28.9)	
Present	-	167 (73.6)	435 (72.5)		-	1,149 (68.6)	1,073 (71.1)	

### Relationship among depressive symptoms, suicidal behaviours and smoking frequency

Table 3 shows the association between smoking status and mental health conditions. Regardless of the age group, the prevalence of depressive symptoms and suicide ideation was higher in NDS than that in NS or DS. Among EA, OR values of NDS were observed with regard to depressive symptoms (OR: 1.52, 95% CI: 1.24–1.86), suicide ideation (OR: 1.56, 95% CI: 1.24–1.97), suicide plan (OR: 1.62, 95% CI: 1.18–2.23) and suicide attempt (OR: 1.75, 95% CI: 1.18–2.59), whereas DS was not related to any of the depressive symptoms or suicidal behaviours. Among LA, NDS was strongly related to depressive symptoms (OR: 1.31, 95% CI: 1.15–1.50), suicide ideation (OR: 1.35, 95% CI: 1.14–1.60), suicide plan (OR: 1.57, 95% CI: 1.21–2.03) and suicide attempt (OR: 1.51, 95% CI: 1.09–2.08). Unlike in EA, DS in LA revealed significant associations with depressive symptoms (OR: 1.22, 95% CI: 1.05–1.42), suicide plan (OR: 1.46, 95% CI: 1.09–1.94) and attempt (OR: 2.24, 95% CI: 1.60–3.14).

**Table 3:** Association between smoking patterns with depressive symptoms and suicidal behaviours (KYRBS, 2016, Korean adolescents aged 12–18 years).

		Early adolescents (n = 30,456)				Late adolescents (n = 29,573)			
		Daily smokers		Non-daily smokers		Daily smokers		Non-daily smokers	
		No, n (%)	OR^a^ (95% CI)	No, n (%)	OR^a^ (95% CI)	No, n (%)	OR^a^ (95% CI)	No, n (%)	OR^a^ (95% CI)
**Depressive Symptoms**^**†**^	No	136 (59.9)	1.0 (ref)	352 (58.7)	1.0 (ref)	1,043 (62.2)	1.0 (ref)	922 (61.1)	1.0 (ref)
	Yes	91 (40.1)	1.14(0.79–1.65)	248 (41.3)	1.52(1.24–1.86)	633 (37.8)	1.22 (1.05–1.42)	587 (38.9)	1.31 (1.15–1.50)
**Suicide Ideation**	No	169 (74.4)	1.0 (ref)	441 (73.5)	1.0 (ref)	1,386 (82.7)	1.0 (ref)	1,219 (80.8)	1.0 (ref)
	Yes	58 (25.6)	1.08 (0.71–1.65)	159 (26.5)	1.56 (1.24–1.97)	290 (17.3)	1.19 (0.98–1.46)	290 (19.2)	1.35 (1.14–1.60)
**Suicide Plan**	No	193 (85.0)	1.0 (ref)	526 (87.7)	1.0 (ref)	1,532 (91.4)	1.0 (ref)	1,380(91.5)	1.0 (ref)
	Yes	34 (15.0)	1.57 (0.92–2.66)	74 (12.3)	1.62 (1.18–2.23)	144 (8.6)	1.46 (1.09–1.94)	129 (8.5)	1.57 (1.21–2.03)
**Suicide Attempt**	No	206 (90.7)	1.0 (ref)	551 (91.8)	1.0 (ref)	1,558 (93.0)	1.0 (ref)	1,422 (94.2)	1.0 (ref)
	Yes	21 (9.3)	1.08 (0.54–2.14)	49 (8.2)	1.75 (1.18–2.59)	118 (7.0)	2.24 (1.60–3.14)	87(5.8)	1.51 (1.09–2.08)

^†^Among the various symptoms of depression, this includes feelings of sadness or hopelessness hampering daily activities for over 2 weeks.

^a^Adjusted for sex, residence, family economic status, paternal education, maternal education, academic achievement, sleep satisfaction, current alcohol drinking, experience of violence, second-hand smoking in family and friends’ smoking status

Table 4 presents the analysis of OR values of NDS with regard to depressive moods and suicidal behaviours compared with NS and DS. Among EA, NDS was associated with an increased likelihood of reporting depressive symptoms (OR: 1.33, 95% CI: 1.09–1.61), suicide ideation (OR: 1.41, 95% CI: 1.13–1.76) or suicide plan (OR: 1.43, 95% CI: 1.05–1.94) than NS. Notably, NDS in EA were more likely to have a higher risk of depressive symptoms (OR: 1.55, 95% CI: 1.06–2.28) compared with DS. Among LA, NDS was more likely to be associated with depressive symptoms (OR: 1.26, 95% CI: 1.11–1.43), suicide ideation (OR: 1.28, 95% CI: 1.09–1.50), suicide plan (OR: 1.43, 95% CI: 1.12–1.82) and suicide attempt (OR: 1.39, 95% CI: 1.03–1.88) compared with NS. No differences were found in the relationship between NDS and depressive symptoms, suicide ideation and plan compared with DS, but the association between NDS and suicide attempt (OR: 0.65, 95% CI: 0.45–0.93) was significantly low compared with DS.

**Table 4:** Association of depressive symptoms and suicidal behaviours with smoking patterns (KYRBS, 2016, Korean adolescents aged 12–18 years).

	Early adolescents (n = 30,456)			Late adolescents (n = 29,573)		
	Never smokers (n = 29,629)	Daily smokers (n = 227)	Non-daily smokers (n = 600)	Never smokers (n = 26,388)	Daily smokers (n = 1,676)	Non-daily smokers (n = 1,509)
**Depressive Symptoms**^**†**^						
No, N	23,252	136	352	19,520	1,043	922
Yes, N	6,377	91	248	6,868	633	587
**OR**^**a**^ **(95% CI)**	**1.0 (ref)**	**0.90 (0.66–1.25)**	**1.33 (1.09–1.61)**	**1.0 (ref)**	**1.17 (1.02–1.34)**	**1.26 (1.11–1.43)**
**OR**^**b**^ **(95% CI)**	**-**	**1.0 (ref)**	**1.55 (1.06–2.28)**	**-**	**1.0 (ref)**	**1.01 (0.85–1.19)**
**Suicide Ideation**						
No, N	26,325	169	441	23,453	1,386	1,219
Yes, N	3,304	58	159	2,935	290	290
**OR**^**a**^ **(95% CI)**	**1.0(ref)**	**0.95 (0.66–1.37)**	**1.41 (1.13–1.76)**	**1.0 (ref)**	**1.16 (0.97–1.38)**	**1.28 (1.09–1.50)**
**OR**^**b**^ **(95% CI)**	**-**	**1.0 (ref)**	**1.52 (0.98–2.35)**	**-**	**1.0 (ref)**	**1.02 (0.82–1.27)**
**Suicide Plan**						
No, N	28,484	193	526	25,562	1,532	1,380
Yes, N	1,145	34	74	826	144	129
**OR**^**a**^ **(95% CI)**	**1.0 (ref)**	**1.20 (0.76–1.91)**	**1.43 (1.05–1.94)**	**1.0 (ref)**	**1.54 (1.19–1.99)**	**1.43 (1.12–1.82)**
**OR**^**b**^ **(95% CI)**	**-**	**1.0 (ref)**	**1.25 (0.73–2.15)**	**-**	**1.0 (ref)**	**0.87 (0.64–1.19)**
**Suicide Attempt**						
No, N	28,953	206	551	25,979	1,558	1,422
Yes, N	646	21	49	409	118	87
**OR**^**a**^ **(95% CI)**	**1.0 (ref)**	**1.01 (0.57–1.79)**	**1.44 (0.99–2.10)**	**1.0 (ref)**	**1.94 (1.43–2.64)**	**1.39 (1.03–1.88)**
**OR**^**b**^ **(95% CI)**	**-**	**1.0 (ref)**	**1.89 (0.96–3.72)**	**-**	**1.0 (ref)**	**0.65 (0.45–0.93)**

^†^Among various symptoms of depression, this includes feelings of sadness or hopelessness hampering daily activities for over 2 weeks.

^a^Adjusted for sex, residence, family economic status, paternal education, maternal education, academic achievement, sleep satisfaction, current alcohol drinking, experience of violence, second-hand smoking in family and friends’ smoking status

^b^Adjusted for sex, residence, family economic status, paternal education, maternal education, academic achievement, sleep satisfaction, current alcohol drinking, experience of violence, second-hand smoking in family and friends’ smoking status, first experience of smoking, smoking volumes and trying to quit smoking during the past 12 months

## Discussion

This study is the first national representative study to demonstrate that non-daily smoking during adolescence is associated with an increased risk for depressive symptoms and suicidal behaviours. The results of this study reflected that non-daily smoking was becoming a behaviour of adolescents who were vulnerable to structural disadvantages [37], such as low household income, low paternal and maternal education level or more experience of violence, and the tendency was remarkable in LA. Our results also suggested that depressive symptoms and suicidal behaviours are significantly associated with adolescent non-daily smokers compared with not only NS but also DS. The results of this study expanded upon and shared similarities with previous findings on the relationship between mental health and non-daily smoking. The prevalence of depressive symptoms of non-daily smokers in this study was 39.5%, which is similar to previous findings [8]. Results of the study is consistent with the previous reports that non-daily smoking is associated with increased risk for depression diagnoses (OR 1.29) [38]. Prior studies suggested that NDS were more likely than DS to emphasize on negative reinforcement motives of smoking [39]and they often smoke when emotionally distressed [40].

We found that the prevalence of non-daily smoking and daily smoking in the present data was considerably lower than that in other studies [41]. In our study, 2.71% of early adolescents and 10.66% of late adolescents had smoked cigarettes daily or non-daily during the past 30 days. The results are consistent with previous study that reported the lower prevalence of Korean adolescent smoking compare to other countries [42]. In addition to low smoking rate of Korean adolescents, these findings may be partly explained by the fact that the survey was self-reported, and adolescents may possibly tend to report less or hide their smoking status.

The fact that differences were found in the association between smoking frequency and mental health in different ages might reflect various mechanisms of smoking, such as genetics, self-medication, incentive learning and motivational processes [43]. In the previous study, non-daily smokers had significantly lower levels of addiction according to the modified Fagerstrom Tolerance Questionnaire and reported fewer smoking volume, which means that non-daily smokers had different vulnerabilities to nicotine dependence compared with daily smokers [9]. Our findings are consistent with the results of a prior study, which state that exposure to smoking at home seems to be an important risk factor for adolescent smoking [44], and the trend was more significant in NDS in our results. However, in our study, non-daily smokers seemed to have fewer number of friends who smoke compared with DS This finding is leading to the assumption that NDS among adolescents is possibly related to mental health problems more than we expected, and smoking was used for self-relief medication (e.g., relieve depressive mood or anxiety, relax from the stressful environment) rather than fitting with the stereotype of non-daily smoking, which is sometimes referred to as ‘social smoking’ in adults [45].

Despite in overall smoking rate continues to decline among adolescents, the decline in non-daily smoking is considerably slower [8]. Depression is a well-known common cause of smoking cessation failure [46], and our results suggested that NDS in EA were more likely to not try to quit smoking during the past year compared with DS. Furthermore, this trend might be explained by the high association between NDS and depression in EA that followed in the study.

Although our results do not imply that the direction of the relationship and mechanisms supporting the relationship between NDS and mental health is not well established, some hypotheses are supporting the relationship. Berg suggested that negative affect regulations of non-daily smokers were associated with greater depressive symptoms among college students [47]. It has been also proposed that nicotine may have direct distress relief effects that do not depend on withdrawal-relief, but actually reduce distress from exogenous sources such as depressive symptoms [43]. Thus, the higher relevance of NDS to depressive symptoms may reflect use of smoking for instrumental purposes, which is more associated with non-dependent smoking [39]. In the reverse prospective, another possible explanation for these results is that non-daily smoking may contribute to decreasing dopamine and γ-aminobutyric acid levels, which have been related to an increased risk for depression as much as those observed in daily smokers or more [48]. There were a number of significant differences in depressive symptoms and suicidal behaviour between DS and NDS by age group. Among EA, we found a high risk of depressive symptoms, suicide ideation and suicide plan for NDS, whereas none of these are associated with DS. NDS also had a significantly higher risk of depressive symptoms than DS, even after controlling for age of first smoking, a volume of smoking and whether they tried to quit smoking during the past year. Interestingly, among LA, despite NDS having higher risks of depressive symptoms and suicide ideation, DS had distinctly higher risks of the suicide plan and suicide attempt compared with NDS. This finding was consistent with the results of prior meta-analyses that cigarette smoking is associated with a significantly increased risk of suicidal behaviours, and that there is an intensity response relationship [49,50]. DS in LA who smoke for a longer period are more likely to be nicotine dependent. The results support the hypothesis that chronic exposure to cigarette smoking is an additional risk factor for suicidal behaviour beyond depressive symptoms [51]. DS among LA, likely to be heavy smoking, also has direct downstream effects on dopaminergic and glutamatergic neurotransmitter systems with roles in impulsivity and decision making that affect suicide plans and attempts [52]. We presumed that as NDS in EA become DS in LA over time [4], NDS in EA had a high risk for depressive symptoms and suicide ideations, but DS in LA had a high risk for suicide plans and attempts. However, this hypothesis should be clarified through longitudinal studies in the future.

A number of potential mechanisms for the association between depression, suicidal behaviours and smoking have been intensively researched in various countries, but few studies have reached a universal consensus to understand their clear relationship [53]. Despite the consistent results that implicated a risk of depression among adolescent smokers [54], the mechanism of association between non-daily smoking and depression among adolescents is still unclear, and further research on the association is needed. This study was based on a large sample size from a nationwide adolescent population survey that has shown good representativeness and generalizability. The results of the present study may suggest the need for intervention in the mental health for non-daily smokers, especially for EA. These findings demonstrate how raising awareness in adolescent non-daily smokers and developing prevention and treatment programmes targeting non-daily smokers in adolescents are important and necessary.

The present study has a number of limitations that should be considered when interpreting the findings. First, as presented, the cross-sectional designs of the present findings do not allow for examinations of how depressive symptoms and suicidal behaviours influence each other. Future studies with sufficient time for follow-up are needed to develop a greater temporal understanding of the relationship between non-daily smoking and suicidal behaviours. Second, this study is based on the use of self-reports, and it may result in the tendency of the subjects to under-report cigarette smoking or suicidal behaviours, and there is also a possibility of information bias. Finally, unmeasured confounding variables that may affect depressive symptoms or suicidal behaviours could not be analysed in this study. Despite the limitations of this cross-sectional survey, the present study has the following strengths. It used a large nationwide representative sample of Korean adolescents. The response rate to this survey was high. Finally, non-daily smoking was investigated as a risk factor for depression and suicidal behaviours with various confounding factors in adolescents. To the best of our knowledge, this is the first research on the association of depressive symptoms and suicidal behaviour with non-daily smoking in adolescents.

## Conclusion

In conclusion, in the present study of Korean adolescents aged 12–18 years, the prevalence of depressive symptoms, suicide ideation, suicide plan and suicide attempt for non-daily smokers was greater than NS. Particularly for EA, those who smoke less and those who appear not to be dependent on nicotine, the prevalence of depressive symptoms of non-daily smokers was greater compared with daily smokers. In addition, the results of the study showed significant differences in smoking-related behaviours between non-daily smokers and daily smokers in adolescents. Therefore, early and tailored preventive measures for this risk group are essential. More researches on the mental health of non-daily smokers are needed to better understand the risk behaviours of this understudied population relative to their importance as a group of adolescent smokers.
